# Liquid-liquid phase separation driven by charge heterogeneity

**DOI:** 10.1038/s42005-024-01875-4

**Published:** 2024-12-19

**Authors:** Daniele Notarmuzi, Emanuela Bianchi

**Affiliations:** 1 Institut für Theoretische Physik, TU Wien, Wiedner Hauptstraße 8-10, A-1040 Wien, Austria; 2 CNR-ISC, Uos Sapienza, Piazzale A. Moro 2, 00185 Roma, Italy

**Keywords:** Biological physics, Phase transitions and critical phenomena

## Abstract

Despite the intrinsic charge heterogeneity of proteins plays a crucial role in the liquid-liquid phase separation (LLPS) of a broad variety of protein systems, our understanding of the effects of their electrostatic anisotropy is still in its early stages. We approach this issue by means of a coarse-grained model based on a robust mean-field description that extends the DLVO theory to non-uniformly charged particles. We numerically investigate the effect of surface charge patchiness and net particle charge on varying these features independently and with the use of a few parameters only. The effect of charge anisotropy on the LLPS critical point is rationalized via a thermodynamic-independent parameter based on orientationally averaged pair properties, that estimates the particle connectivity and controls the propensity of the liquid phase to condensate. We show that, even though directional attraction alone is able to lower the particle bonding valence—thus shifting the critical point towards lower temperatures and densities—directional repulsion significantly and systematically diminishes the particle functionality, thus further reducing the critical parameters. This electrostatically-driven shift can be understood in terms of the additional morphological constraints introduced by the directional repulsion, that hinder the condensation of dense aggregates.

## Abstract (web summary)

Experiments show that charge heterogeneity in proteins affects their liquid-liquid phase separation (LLPS). Using a theoretically grounded and numerically efficient coarse-grained model, the authors study how the amount of charge and its surface distribution affects the LLPS. They find that electrostatics controls the connectivity of particles thus impacting the emergence of the LLPS.

## Introduction

The relevance of electrostatics for the liquid-liquid phase separation (LLPS) of proteins has been highlighted by a large number of experiments on globular protein systems, such as lysozyme, *γ*- and *β*-crystalline, bovine pancreatic trypsin inhibitor, thaumatine, human hemoglobin, and glucose isomerase, where the condensation of a dense phase was shown to be sensitive to variations of pH and salt concentration^1–7^. Although the electrostatic interactions driving LLPS in these systems have been regarded as isotropic^8,9^, charge anisotropy is inherently unavoidable due to the clustering of amino acids with similar physicochemical properties, leading to the formation of positive and negative surface patches. The features of these patches depend on the local environment—which governs the protonation state of the ionizable groups along the protein backbone—and vary significantly among different proteins^10–14^.

Recent experiments demonstrate that charge patchiness can be exploited to control LLPS in a wide range of protein systems. For instance, the precise engineering of globular protein mutants enables to trigger their complexation with oppositely charged polyelectrolytes at desired pH values, even at those pH values where proteins are neutral^15^. This was achieved by tuning the protein surface charge distribution—characterized by a number of patches ranging typically from one to three—while keeping the net charge fixed. Moreover, a different set of experiments investigated the role played by the size of these surface patches: mutants with larger charged patches were shown to more readily form complexes in comparison to mutants with smaller patches^16^.

Electrostatic anisotropy has further been shown to affect the LLPS of homo- and hetero-molecular systems of antibodies. In the particular case of hetero-molecular systems, the non-specific affinity of a library of antibodies—tested via their assembly mechanism with single-stranded DNA ligands—was recently proven to be driven by charged patches in the low salt regime, where hydrophobic patches play a minor role. Under these conditions, LLPS emerged as a result of electrostatic interactions, whose effects were related to the size of the surface patches^17^.

Furthermore, charge patchiness can be taken advantage of even to regulate the LLPS of intrinsically disordered proteins (IDPs), a phenomenon which is notoriously due to specific and multivalent interactions between the disordered domains^18,19^. A particularly recent investigation targeting the LLPS of small synthetic proteins has shown that, while the phenomenon emerges due to the multivalent interactions between disordered regions, the pH-dependence of the proteins solubility derives from the change in the protonation state of surface amino acids, thus indicating that the surface patchiness of folded modular domains can be exploited to tune the LLPS by means of pH changes^20^.

These findings suggest that engineering non-specific interactions through charge heterogeneity can enhance control over the LLPS of both globular and disordered proteins. This leads to the timely and fundamental question of how features such as patch size, net particle charge, and charge asymmetry influence the LLPS and whether anisotropic electrostatics alone is able to drive this process.

In the present work, we aim to address these issues by considering a minimalistic coarse-grained model featuring spherical particles with orientational-dependent interactions stemming purely from the charge patchiness of the proteins^21,22^. This model was first introduced to describe micron-scale colloids with heterogeneous surface charge distributions^23–27^ and builds up on extending the standard mean-field theory developed for uniformly charged objects in a dilute electrolyte to non-uniformly charged ones.

In this respect, it is worth noting that coarse-grained models for proteins rooted in colloid physics have been able to provide crucial insights on some fundamental features of the LLPS observed in protein systems^28,29^. In particular, spherical models with isotropic, short-ranged interactions have shed light on the metastability of the LLPS in globular protein systems^30–33^ and on their crystallization mechanisms^34,35^. Furthermore, hard-sphere models with a limited number of functional bonding sites—mimicking the multi-valency of the proteins binding groups—show that on reducing the average valence of the systems the drive for the condensation of the dense phase is drastically reduced^36–39^. Experimental data are nowadays often rationalized using the reduced functionality of globular proteins as a control parameter of their LLPS^7,40–43^, their crystallization^44–46^ and gelation mechanism^47^.

Building on these previous achievements, our modeling approach is able to account for both the short-ranged nature of the protein-protein interactions as well as for their bonding anisotropy while taking into account the patchiness of the surface charge distribution. Owing to its relative simplicity, our model can be exploited in statistically robust Monte Carlo simulations and allows us to systematically investigate the effect of the patch size and charge imbalance by varying these features independently and with the use of a limited number of parameters. Our findings reveal that charge patchiness affects the LLPS not only by virtue of the directional attraction between oppositely charged surface regions but also, and most significantly, thanks to the directional repulsion between equally charged regions. They additionally show that a limited bonding valence—usually associated only with specific interactions—can also emerge in non-specific interactions due to the interplay between the position of the patches and the amount of charge associated to each. Despite our model being more suitable for globular rather than disordered proteins, the emerging trends highlighted in our investigation can be related to particle connectivity, thus providing a more general understanding of the impact of anisotropic electrostatics on the LLPS of proteins.

## Results and discussion

### Mean-field electrostatic interactions

In the context of the LLPS, globular proteins have been successfully modeled by the DLVO theory^30,31^, which is a broadly accepted mean-field description of the interactions between homogeneously charged spherical objects in an electrolytic solution^48,49^. Our coarse-grained model is rooted in an extension of such a theoretical framework that has been developed to take into account non-homogeneities in the surface charge distribution of the particles^21,22^. The idea behind this DLVO-like mean-field approach^21,22^ is to reproduce the surface charge density by means of a few effective charges^50–52^, leading to a multipole expansion that extends beyond the monopolar term^12,53^.

**Fig. 1:**
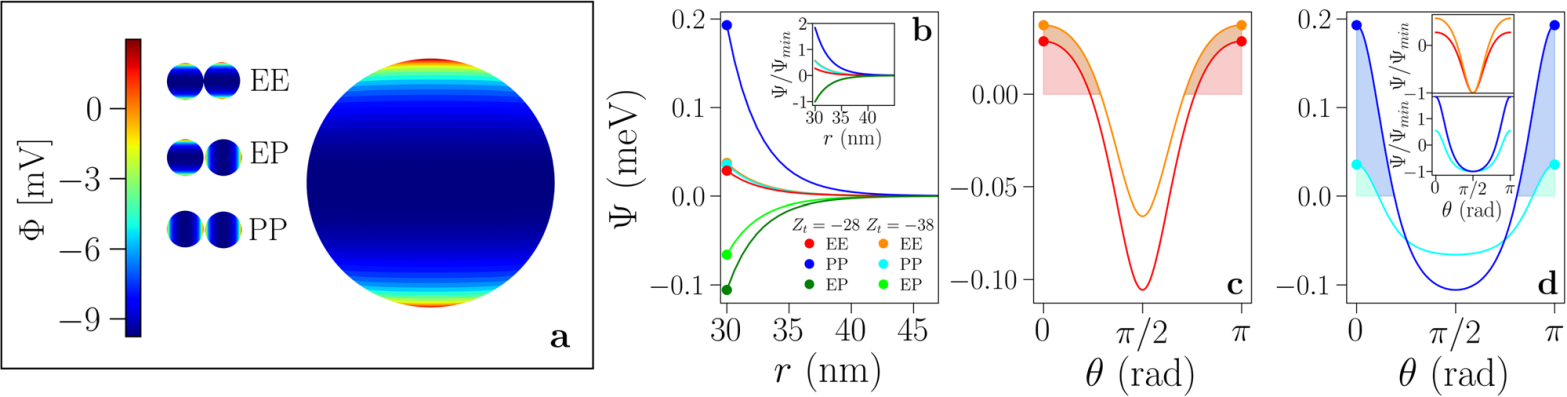
Electrostatics of heterogeneously charged spheres with a null dipole moment and a non-zero linear quadrupole. We consider a particle of diameter 2*σ*_*c*_ = 30 nm in water at room temperature in physiological conditions resulting in *λ*_*B*_ = 0.71 nm and *λ*_*D*_ = 3 nm; we fix *a* = 0.26*σ*_*c*_. **a** Representation of the electrostatic surface potential Φ (in mV) on the particle surface. **b** Interaction energy *Ψ* as a function of the inter-particle separation (in nm) for particles with two different values of the charge imbalance *Z*_*t*_ (in units of the elementary charge), as labeled. Three reference configurations—labeled EE, PP, and EP, and shown in **a**—are considered for each system. Inset: same as in the main, where the energies are normalized with respect to the minimum energy, *u*_EP_. **c** Interaction energy at contact on rotating the particle symmetry axis from the EE, to the EP and back to the EE configuration. **d** Same as in **c** but for the rotation from the PP, to the EP back to the PP configuration. Inset top/bottom: same as in the main of **c**/**d**, where the energies are normalized with respect to the minimum energy, *u*_EP_.

We consider spherical units with a relatively simple surface charge distribution consisting of a negative equatorial belt and two positive, identical polar regions (Fig. 1a). It is worth noticing that the same interaction pattern would emerge by considering a positively charged equator and two negatively charged poles. In the DLVO-like description, this triblock pattern is created by a set of three effective charges: one in the particle center, associated to the equatorial belt, and two off-center sites associated to the polar caps. In the limit of thin double layers and under the assumption that the off-center charges are at the same distance from the center and placed opposite to each other with respect to it—producing an axially symmetric, linear quadrupole—the mean-field interaction can be analytically determined for any set of microscopic parameters^21^. The potential between particles with more complex surface charge distributions can be derived numerically, for both thin and thick double layers, and there is no need for any assumption on the charge positions^22^. The microscopic parameters entering this effective pair potential are the radius of the particles, *σ*_*c*_, the Bjerrum length *λ*_*B*_, the Debye length *λ*_*D*_, the distance between the off-center charges and the central one, *a* < *σ*_*c*_, and the valence of the three effective charges, *Z*_*i*_, with *i* = 1, 2, 3. The analytic pair interaction potential, *Ψ*, has the form of the standard screened Coulomb potential of the DLVO theory, multiplied by a non-isotropic term which retains the symmetries of the single particle potential, Φ, thus accounting for the contributions of the off-center charges^21^. We show in Fig. 1 the pair potential of two model systems with identical sets of microscopic parameters differing only in the net particle charge *Z*_*t*_ = ∑_*i*_ *Z*_*i*_. The radial dependence of the pair energy (Fig. 1b) is reported for three configurations: the equator-equator (EE), the pole-pole (PP), and the equator-pole (EP) orientation (sketched in Fig. 1a). We label the energies at the contact of these three configurations as *u*_EE_, *u*_PP_ and *u*_EP_, respectively. As highlighted by the behavior of the curves normalized by the system energy minimum, *u*_EP_, on changing *Z*_*t*_ the ratio between *u*_EE_ and *u*_PP_ varies (inset of Fig. 1b): for a relatively small *Z*_*t*_ the PP repulsion dominates over the EE one, while for a larger *Z*_*t*_ the two repulsive contributions become comparable. At fixed interaction range and for a given surface pattern, we can thus characterize different systems with the set of **u** = {*u*_EE_, *u*_EP_, *u*_PP_} (expressed in units of *u*_EP_). If **u** is expressed in units of *u*_EP_, the model system labeled as *Z*_*t*_ = −28 (in units of elementary charge) is identified by **u** = {0.27, −1.00, 1.83} and the model system labeled as *Z*_*t*_ = −38 by **u** = {0.57, −1.00, 0.54}. The direction-dependence of the pair interaction is represented for subsets of possible rotations and taking advantage of the cylindrical symmetry of the surface pattern (Fig. 1c, d). The mixed attractive/repulsive character of the mean-field potential clearly emerges from such a representation, where the relative weight of the positive (shaded areas) and negative contributions to the pair energy depends on the chosen microscopic parameters. The coarse-grained model we propose in the following preserves the (radial and angular) behavior dictated by the charge imbalance while offering fine control over the potential by means of a few independent parameters.

### Coarse-grained model of effective interactions

**Fig. 2:**
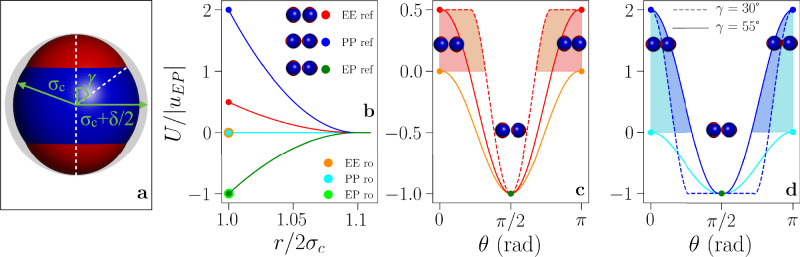
Inverse Patchy Particle (IPP) and their interaction potential. **a** IPP model representation: the blue sphere is the hard core particle, the red polar caps depict those portions of the interaction spheres associated to the off-center sites that protrude out of the particle, the gray shade represents the interaction sphere associated with the central site. Note that IPPs are spherical units. The geometrical parameters of the IPP model are highlighted: the particle radius, *σ*_*c*_ = 0.5, the particle interaction radius, *σ*_*c*_ + *δ*/2, and the half-opening angle, *γ*, as labeled. In this example *γ* = 55°. **b** Interaction energy at any *γ* as a function of the inter-particle separation for pairs of particles in three reference configurations—labeled as EE, PP, and EP, shown on the side of the legend—for IPPs with *u*_EE_ = *u*_PP_ = 0.0 (ro) and for IPPs with *u*_EE_ = 0.5, *u*_PP_ = 2.0 (ref). Note that the orange and cyan lines coincide. **c** Interaction energy at contact (*r* = 2*σ*_*c*_) on rotating the particle symmetry axis from the EE, to the EP and back to the EE configuration for *γ* = 55° (solid) and for *γ* = 30° (dashed). The orange line is for *u*_EE_ = 0.0, the red lines are for *u*_EE_ = 0.5. The shaded area spans configurations with 0 < *U*/|*u*_EP_| < 0.5, highlighting how the interaction energy depends on both energetic and geometrical parameters. **d** Same as in **c** but for the rotation from the PP, to the EP back to the PP configuration. The cyan line is for *u*_PP_ = 0, the blue lines are for *u*_PP_ = 2. The shaded area spans configurations with 0 < *U*/|*u*_EP_| < 2.

The patchy model proposed here is devised as a computationally efficient coarse-grained description mimicking its mean-field counterpart^21,22^. Particles are hard spheres of diameter 2*σ*_*c*_ = 1 and the triblock pattern emerges from a set of three interaction sites: the central one associated with the equatorial belt and two off-center ones associated with the polar caps, also referred to as patches (Fig. 2a). The interaction radius *σ*_*p*_ of both off-center sites is constrained by *σ*_*p*_ + *a* = *σ*_*c*_ + *δ*/2, where *σ*_*c*_ + *δ*/2 is the interaction radius of the central site and *δ* defines the particle interaction range. This constraint—imposed by the screening conditions of the solvent—implies that the half-opening angle, *γ*, describing the patch surface area is $\gamma =\arccos [({\sigma }_{c}^{2}+{a}^{2}-{\sigma }_{p}^{2})/2a{\sigma }_{c}]$ (see Fig. 2a). We fix *δ* = 0.2*σ*_*c*_ and vary *γ* between 30° and 55°.

The coarse-grained inter-particle potential relies on the idea that different contributions stemming from like-charged/oppositely charged regions can be factorized into a characteristic energy strength and a geometric weight factor. The first accounts for the characteristic interaction energies between differently charged surfaces, and the latter accounts for the distance *r* and the particles mutual orientation Ω. Namely, the interaction energy between two particles is infinitely repulsive for distances *r* < 2*σ*_*c*_ and zero for *r* > 2*σ*_*c*_ + *δ*, otherwise, it is a weighted sum of energy contributions:

1 $$U(r,\Omega )={\sum}_{\alpha \beta }{\epsilon }_{\alpha \beta }{\omega }_{\alpha \beta }(r,\Omega )$$

where *α* and *β* specify the interaction sites (central or off-center), *ω*_*αβ*_ accounts for the geometric weight of the *αβ* contribution to the pair energy, and *ϵ*_*αβ*_ characterizes the energy strength of the *αβ* interaction. The *ω*_*αβ*_-values are proportional to the overlap volume between all pairs of interaction spheres pertaining to the *αβ* interaction^21^. While the *ϵ*_*αβ*_-values can be fixed by mapping the coarse-grained potential to the DLVO-like potential^21,22^, here we change them arbitrarily by fixing the set **u** and inverting the three-by-three system of equations resulting from Eq. (1)– see Supplementary Note 1 (SN 1) for details. At fixed interaction range and patchiness, varying the set **u**—and, with it, the *ϵ*_*αβ*_—is equivalent to changing the net particle charge, for instance by tuning the pH of the solution. For each patch size, we consider systems with different **u** to systematically explore the role of directional electrostatic interactions on the LLPS. It is worth stressing that, while we do not target any specific systems, we expect that the chosen model parameters cover a large spectrum of combinations of microscopic parameters. As we need a reference attraction energy in order to compare results from different systems in a meaningful way, we fix *u*_*EP*_ = −1.0 and vary the ratio *u*_*PP*_/*u*_*EE*_ only, thus focusing on the role of the directional electrostatic repulsion. Our choice of reduced units implies that when mapping the coarse-grained systems back to the physical ones, both the size and the charge of the particles can be varied to match a specific set of values (*u*_EE_, *u*_EP_, *u*_PP_) characterized by the target *u*_*PP*_/*u*_*EE*_ ratio. Figure 2 shows characteristic representations of *U*/|*u*_EP_| as a function of the inter-particle distance, panel (b), and of the mutual orientation at contact, panels (c, d), for two different energy sets **u** = {0, −1, 0} and **u** = {0.5, −1, 2} at the largest (*γ* = 55°) and the smallest (*γ* = 30°) patch size. We refer to Inverse Patchy Particles (IPPs) with any *γ* and these two energy sets as, respectively, IPP_ro_ (repulsions off)—as both EE and PP repulsions are zero—and IPP_ref_ (reference)—as the ratio *u*_*PP*_/*u*_*EE*_ = 4.0 was observed in other IPP systems^21,22,54,55^. The selected systems fairly reproduce the behavior of the two mean-field systems proposed in Fig. 1, thus guaranteeing a qualitative connection to the macroscopic physical parameters characterizing this kind of system.

### Effect of the directional attraction

**Fig. 3:**
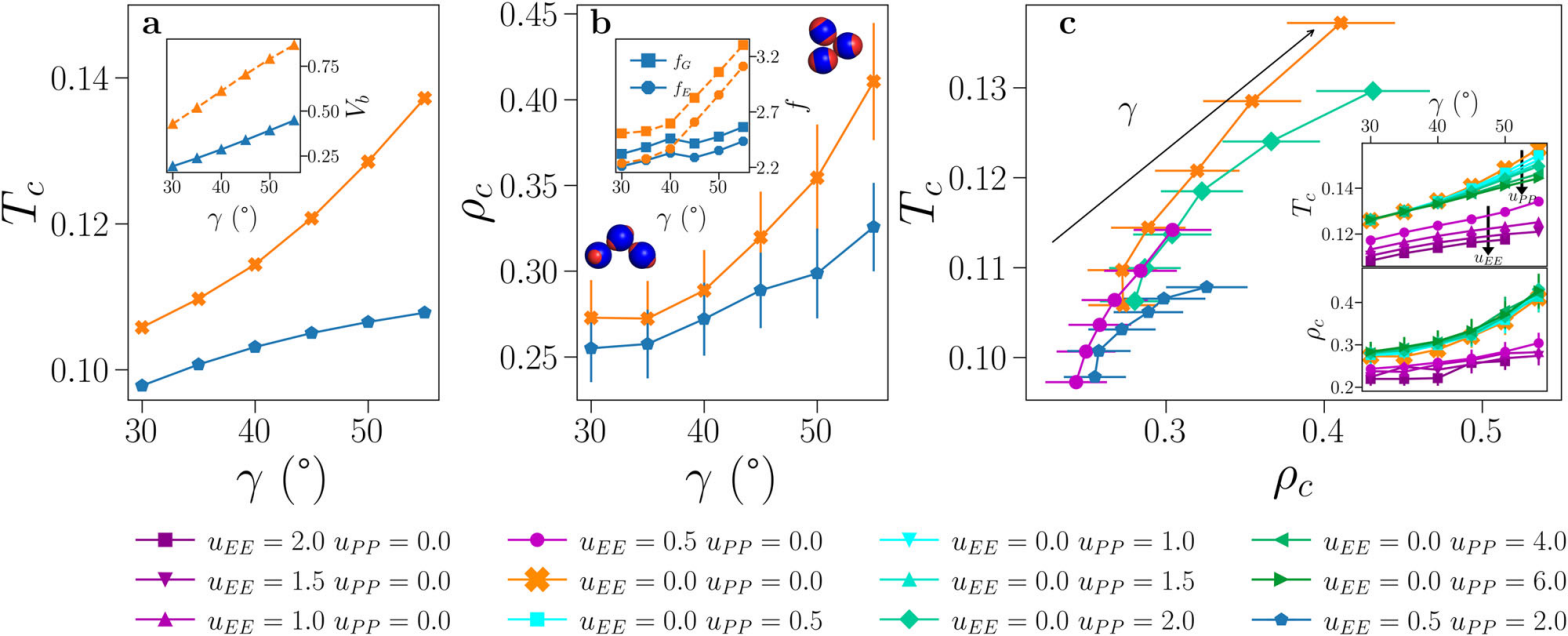
Critical behavior overview. **a** Critical temperature, *T*_*c*_, and **b** critical density, *ρ*_*c*_, as functions of *γ*, for IPP_ro_ (orange) and IPP_ref_ (blue). **a** bonding volume *V*_*b*_ as a function of *γ*. **b** Geometric and energetic functionality (*f*_*G*_ and *f*_*E*_, respectively). **b** further shows two typical trimers for *γ* = 30° (bottom left) and *γ* = 55° (upper right), sampled at the critical point of the IPP_ro_ system. **c** Critical point behavior in the *T*−*ρ* phase diagram for IPP_ro_ and IPP_ref_ systems, as well as for systems with *u*_*EE*_ = 0.0, *u*_*PP*_ = 2.0 and *u*_*EE*_ = 0.5, *u*_*PP*_ = 0.0. Inset of **c** *T*_*c*_ (top) and (bottom) *ρ*_*c*_ versus *γ* on increasing either the EE or the PP repulsion with respect to the IPP_ro_ systems. Errorbars on the critical density correspond to the standard deviation of the density distribution, see SN 2C for further information.

IPP_ro_ systems are considered first as they allow us to isolate the role played by the patch extension *γ*. Both the critical temperature *T*_*c*_ and the critical density *ρ*_*c*_, Fig. 3a, b, respectively, decrease monotonically as *γ* decreases, implying that the LLPS region shrinks on reducing the patch size.

Previous studies suggest that the LLPS moves to lower temperatures and densities when the particle bonding valence—i.e., the maximum number of bonded nearest neighbors—is reduced by in-built particle features^38,39,42,56^. In the context of conventional patchy colloids, this valence can be quantified by the average number of patches per particle—if each patch can form only one bond—or by the percentage of particle surface covered by the patches—i.e., by the patch size^38,56^. In contrast, the valence of IPPs cannot be easily related to the number of patches, as the one bond per patch condition is never guaranteed, nor to their surface extension, because of the complementary nature of the interactions: small patches offer little volume to a large equatorial region, while large patches can form bonds only with thin equatorial belts. This conundrum is resolved by directly measuring the amount of volume available to bonding for a pair of IPPs.

Two particles are defined to form a geometric bond, *G*_*b*_, if they are within their interaction distance (2*σ* ≤ *r* ≤ 2*σ* + *δ*). The bonding volume, *V*_*b*_, is calculated by evaluating the amount of physical volume available to form geometric bonds with negative energy for any configuration of two interacting particles (see SN 3B). Figure 3a (inset) shows *V*_*b*_ as a function of *γ*: consistently with the behavior of *T*_*c*_, it monotonically increases with *γ*, implying that the number of energetically bonded configurations of a pair of particles grows continuously with the size of the polar caps even for significantly reduced equatorial regions.

The behavior of *V*_*b*_ suggests that the average functionality of the particles, *f*, should also monotonically grow with *γ*. We calculate *f* as the average number of bonds per particle in the simulations, distinguishing between geometric and energetic bonds, *f*_*G*_ and *f*_*E*_ respectively (Fig. 3b, inset). Note that to evaluate these quantities the critical point must be known with high precision and simulations must be performed exactly at the inferred critical point (see SN 2C). It must also be noted that while the functionality of conventional patchy colloids is often associated with the number of attractive sites and is thus independent of the thermodynamic conditions, here, *f* is a state-dependent quantity. Moreover, as *f* is sampled in both the dilute and dense phases, it experiences large fluctuations around its mean value. Consistently with the behavior of *V*_*b*_, both *f*_*G*_ and *f*_*E*_ monotonically grow with *γ*, meaning that—despite the complementarity of polar and equatorial regions—the average bonding valence of the system always grows with the patch size, similar to conventional patchy colloids.

**Fig. 4:**
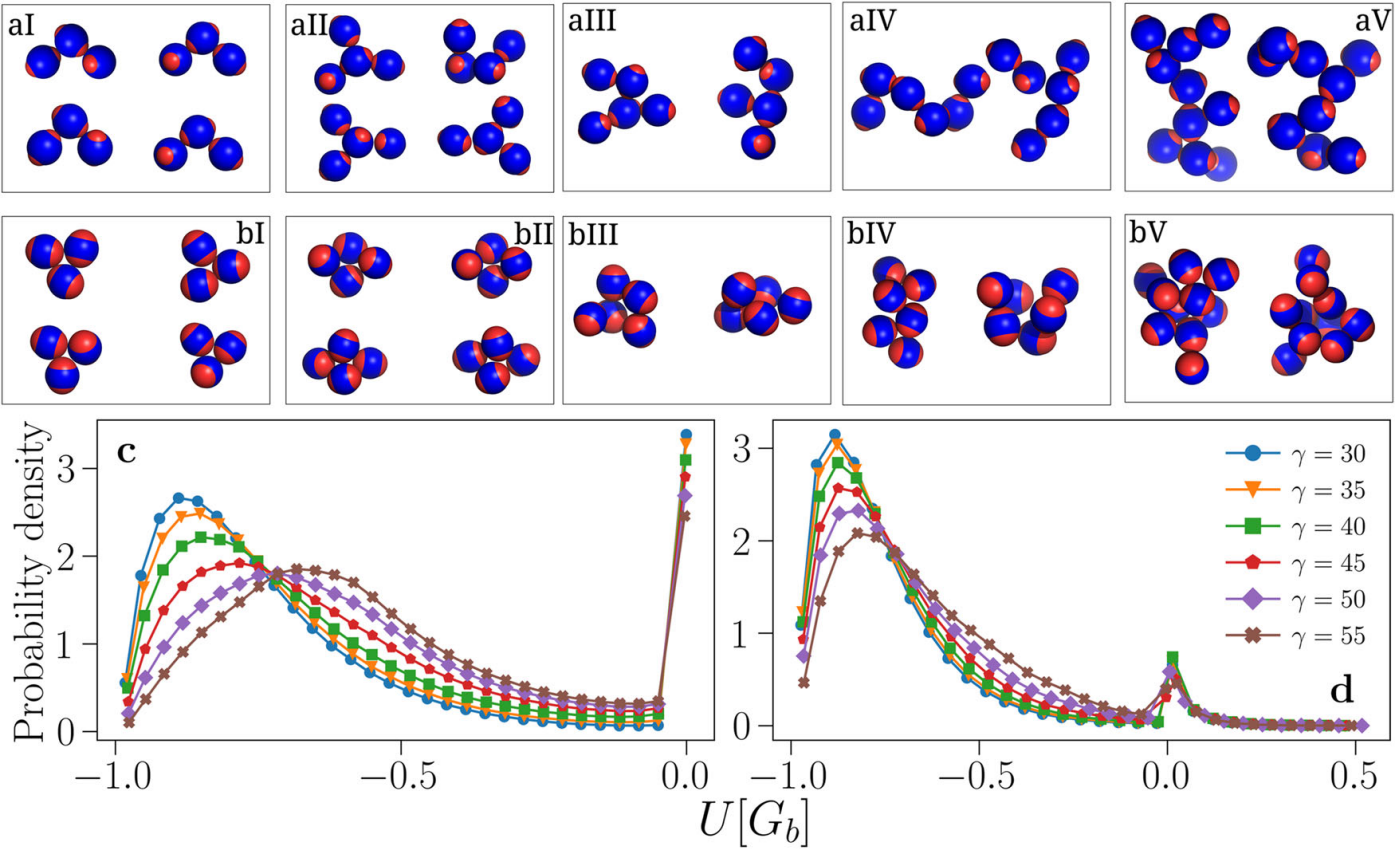
Particles arrangement in small clusters and pair energy distribution. **a**I–**b**V Clusters sampled at the critical point. In the representation of the particles, the blue spheres are the hardcore particles and the red polar caps depict those portions of the interaction spheres associated with the off-center sites that protrude out of the particles. **a**I–**a**V IPP_ro_ systems with *γ* = 30°. **b**I–**b**V IPP_ro_ systems with *γ* = 55°. From I to V, for both line (**a**) and line (**b**), clusters of size 3, 4, 5, 6, and 10. **c** Probability distributions of the interaction energy of geometrically bonded pairs of particles for configurations sampled at the critical point for IPP_ro_. **d** Same as in **c** but for IPP_ref_ systems. Snapshots of the whole system at the critical point are shown in the SN 3A for two IPP_ro_ systems with different values of *γ*.

The reduction of *V*_*b*_ is also accompanied by structural changes in the liquid phase: Figure 3b shows two trimers obtained from samples at the critical point as examples of structures observed in the simulations: a branched trimer formed by IPPs with *γ* = 30° (lower left) and a compact trimer formed by IPPs with *γ* = 55° (upper right). While for large *γ*-values both compact and branched trimers are observed, for small *γ*-values compact trimers are almost entirely absent. These considerations extend beyond the simple case of trimers and can be made quantitative by measuring the average radius of gyration of the emerging clusters^57^ (see SN 3A). Structures ranging from timers to large clusters are, in general, more compact at large *γ*, meaning that branched structures—i.e., less dense clusters—become increasingly rare for large patch sizes (or, equivalently, for thin equatorial belts). On decreasing *γ* (or, equivalently, on increasing the equatorial belts), less compact structures emerge, *ρ*_*c*_ reduces, and the resulting liquid phase is reminiscent of the “empty liquid” observed for conventional patchy colloids. A visual inspection of the systems further supports this analysis: Fig. 4 shows clusters of size 3,4,5,6 and 10 sampled at the critical point for two IPP_ro_ systems, one with *γ* = 30° and one with *γ* = 55°. When patches are small (panels aI–aV), clusters have a particularly branched structure as particles tend to avoid contacts between equatorial regions in order to maximize the EP interactions. In the system with large patches, branched configurations are still present (not shown), but compact structures—as the ones shown in panels bI–bV of Fig. 4—emerge. The existence of these compact structures is guaranteed by the fact that particles can orient themselves so as to have a single patch interacting with more than one equatorial region at the same time, a possibility that cannot occur for small patches.

In summary, in absence of directional repulsive interactions, IPPs with small patches have a small bonding volume—leading to a low *T*_*c*_—and an associated reduced functionality, which supports the formation of branched clusters—leading to a low *ρ*_*c*_.

### Role of electrostatic repulsion

The role of the electrostatic repulsion can be assessed by considering IPP_ref_ systems: in this case, both *u*_*EE*_ and *u*_*PP*_ are different from zero, with *u*_*EE*_ << *u*_*PP*_, as observed in many inverse patchy colloid systems. We report the corresponding *T*_*c*_ and *ρ*_*c*_ as a function of *γ* in Fig. 3a and b, respectively. The behavior of both critical parameters is again monotonically decreasing with decreasing *γ*, where the effect of the directional electrostatic repulsion is to significantly reduce both *T*_*c*_ and *ρ*_*c*_ with respect to the IPP_ro_ systems. This electrostatically-driven shift towards even lower temperatures and densities is *γ*-dependent: for small patches, the change with respect to the IPP_ro_ counterpart is about 8% in *T*_*c*_ and 12% in *ρ*_*c*_, while for large *γ*-values, it raises up to about 27% and 32% respectively. Consistently with the previous analysis, *V*_*b*_ remains monotonically growing with *γ*, but it decreases by more than 50% with respect to the IPP_ro_ systems (Fig. 3a, inset). Similar to the behavior of the critical parameters, the electrostatically-driven reduction of *V*_*b*_ grows with *γ*. The functionalities *f*_*G*_ and *f*_*E*_ also decrease when electrostatic repulsion is present (Fig. 3b, inset) by a strongly *γ*-dependent factor that is negligible for small patches and is particularly large for large patches, mirroring the behavior of the bonding volume as expected.

To better visualize the dramatic effect electrostatics has on the liquid-liquid critical point, we display *T*_*c*_ and *ρ*_*c*_ in the *T*−*ρ* phase diagram for both IPP_ro_ and IPP_ref_ systems (Fig. 3c). This representation clearly shows that, beyond a reduction of both *T*_*c*_ and *ρ*_*c*_ when the electrostatic repulsion is on, the range spanned by these two parameters shrinks as *γ* grows, indicating that geometry becomes less crucial when it interplays with electrostatics. In the same figure, we report the critical point of two additional systems interpolating between the ones already discussed, where *u*_EE_ and *u*_PP_ are varied independently. We observe that, while both directional repulsions are able alone to disfavor the condensation of the liquid phase, the effect of a slight increase of the equatorial repulsion (from zero to 0.5) is stronger than a much larger increase of the polar repulsion (from zero to 2).

Results obtained by further varying *u*_EE_ and *u*_PP_ individually are shown in the insets of Fig. 3c, upper and lower panel, respectively. The critical temperature and density remain monotonically increasing with *γ*. We observe that at any *γ* *T*_*c*_ decreases significantly as *u*_EE_ grows, while the effect of increasing *u*_PP_ is significant only at large *γ*-values and for *γ* = 30° *T*_*c*_ only slightly grows with *u*_PP_. The behavior of *T*_*c*_ is in good agreement with the behavior of the bonding volume (see SN 3B): *V*_*b*_ decreases as electrostatic repulsion grows, with *u*_EE_ having more effect than *u*_PP_. The EE repulsion has a stronger effect than the PP repulsion also for *ρ*_*c*_. In this case, *ρ*_*c*_ increases as *u*_EE_ grows, while it (slightly) increases with *u*_PP_, implying that it is the EE repulsion that mostly leads to the formation of a particularly dilute liquid. Notably, the highest *T*_*c*_ is >40% larger than the lowest, and the largest *ρ*_*c*_ is almost 80% larger than the smallest, evidencing the strong effect of the electrostatic repulsion.

Overall, the collected results suggest that the patch-patch repulsion plays a minor role in the critical behavior with respect to the equator-equator repulsion. We argue that this outcome can be related to the reduced number of configurations where the patch-patch interactions between pairs of IPPs actually occur. This is already suggested by the cluster snapshots shown in panels (a) and (b) of Fig. 4 where the alignments of patches belonging to different particles are significantly less than configurations where equators belonging to different particles face each other. In order to be more quantitative, we estimate the probability distribution of the interaction energy of pairs of bonded particles measured in the simulations at the critical point, for both IPP_ro_ and IPP_ref_ systems—at any *γ*-values.

We report in panels (c) and (d) of Fig. 4 the probability distribution of the pair energies *U*[*G*_*b*_]. We observe that, when the electrostatic repulsion is off (IPP_ro_ systems), panel (c), the distribution of bonded energies has two peaks: one at zero and one at a negative energy value, which moves from *U*[*G*_*b*_] = −0.89 to *U*[*G*_*b*_] = −0.68 on increasing *γ* (while the peak broadens). This is compatible with the qualitative discussion about the morphology of the observed clusters: at small patch sizes, particles can better optimize their relative orientation to maximize the EP bonds, while on increasing the patch size both branched and compact clusters emerge, the latter introducing energy penalties due to equator-equator interactions, thus leading to larger *U*[*G*_*b*_]-values. When the electrostatic repulsion is fully on (IPP_ref_ systems, panel (d)), we observe that the amount of configurations with negative bond energy increases with respect to the IPP_ro_ case (at all *γ*s), while the number of configurations with zero bond energy decreases. This effect is due to the additional constraints introduced by the directional repulsion: mutual orientations with non-negative energy are now energetically penalized, while in the absence of directional repulsion, they were effectively cost-free. As a consequence, there is only a small amount of configurations where the electrostatic repulsion plays a role and among those, the number of configurations with *U*[*G*_*b*_] > 0.5 is negligible. As particles are able to self-organize into configurations where the patch-patch repulsion is completely avoided, the patch-patch repulsion is rarely relevant for the critical behavior.

## Conclusions

With the aim of gaining insight into the role of electrostatics in the LLPS of protein systems, we consider a mean-field theory describing the electrostatic interactions between heterogeneously charged globular units. The theory can be regarded as an extension of the DLVO theory for homogeneously charged spheres, which has been proven to be a insightful hypothesis for understanding the phase diagram of globular proteins. The extension used in this work allows for a more realistic description of systems as globular proteins and colloids. A simple, computationally efficient coarse-grained model is considered whose interaction potential mimics the DLVO-like theory for heterogeneously charged systems and is sufficiently simple to allow for large-scale numerical simulations to be performed.

Particles in the coarse-grained model feature orientation-dependent interactions with both directional attraction and directional repulsion, mimicking the opposite-charge and like-charge contributions to the pair energy between heterogeneously charged particles. Despite a one-to-one mapping from the mean-field to the coarse-grained parameters can be guaranteed within our approach, we choose to systematically vary the parameters of the coarse-grained model to understand how each impacts the LLPS. We stress that several microscopic systems fall within the chosen parameter space.

The critical point of the model is systematically investigated upon changing the net particle charge and the charge distribution geometry independently of each other. It is worth stressing that, while the LLPS in globular proteins is often described by means of particle models with a limited bonding valence, in our class of systems, the bonding valence is not an in-built feature of the model but rather emerges as a consequence of the complex interaction pattern.

We report how the geometry of the surface charge distribution and the charge imbalance (or, equivalently, the net particle charge) affect the location of the critical point in the phase diagram. Regarding the effect of the geometry, we find out that the directional attraction emerging from the patchiness of the surface patterns already reduces the particle bonding valence with respect to isotropically interacting systems, thus bringing the critical point towards lower temperatures and densities; this is consistent with the “empty liquid” scenario presented for conventional patchy colloids^38^ and supports the metastabilty of the LLPS of globular proteins^39^. As the charge imbalance controls the ratio between the EE and the PP repulsion, we investigate the effects of the net particle charge by independently varying these two repulsions: we find that the critical point is by far more affected by the EE repulsion, rather than by the PP one. We argue that this is possibly due to the reduced number of configurations where the patch-patch interactions between pairs of IPPs occur.

On top of the reduction of the bonding valence induced by the directional attraction, the directional repulsion further and significantly reduces the particle functionality. As the number of bonded pair configurations diminishes with the directional repulsion, an electrostatically-driven shift of the critical point to lower temperatures and densities is introduced, thus significantly disfavoring the condensation of the liquid phase.

Despite the investigated surface charge pattern—with a null monopole moment and a non-zero linear quadrupole moment—is an unlikely configuration for a globular protein, our results are grounded in the effect that electrostatics has on the particle connectivity. Hence, if more complex charge distributions were implemented by, e.g., increasing electrostatic repulsion (at fixed geometry) or decreasing the volume of repulsive regions (at fixed net particle charge), the particle connectivity (and hence the critical temperature) would still diminish and support the formation of low-density aggregates. A further assessment of the suitability of our model to describe the LLPS of various protein systems comes from the second virial coefficient at the critical point, ${b}_{2}^{* }({T}_{c})$^58^. In fact, ${b}_{2}^{* }({T}_{c})$ of the IPP systems considered here span a range of values that are compatible with experimental observations made on several globular proteins^6,9^ as well as with those recently reported in experiments on folded domains of IDPs^20^ and monoclonal antibodies^59,60^. This striking agreement further stresses the predictive power of our modeling approach well beyond the spherical approximation. An additional indication of the generality of our results is provided by comparison to the critical behavior of block polyampholytes. Recent studies of IDP models composed of sequences of charged beads have demonstrated that increasing charge blockiness—i. e., increasing the patch size, in our language—results in a shift of the critical point towards higher temperatures^61–63^. Furthermore, also in these systems, charge repulsion has been observed to decrease the local density of the condensates and lead to more porous dense phases, as observed in our systems on specifically increasing the directional repulsion. It is worth noticing that going beyond the spherical assumption—by, e.g., considering soft particles or ensembles of charged patchy spheres—would not allow us to take straightforward advantage of the available mean-field description when building the coarse-grained model for large-scale simulations.

The possibility to suppress further and further the LLPS by taking advantage of the electrostatically-driven trends observed in our study is directly relevant, e.g., for protein purification: phase separation can in fact be an issue when pure batches of proteins are needed, for instance, for the accurate determination of their biochemical and biophysical properties or for the in vitro study of their collective behavior^64,65^. The control over protein aggregation is also crucial in biomedical applications as the immune response to aggregated proteins leads to potentially lethal adverse effects^66,67^. Given the large amount of techniques to modify the surface charge distributions both in vivo and in vitro, our investigation suggests that the complex interplay between surface charge heterogeneity and net particle charge provides a road-map to improve strategies in biotherapeutics.

## Methods

### Grand canonical Monte Carlo simulations

The critical points were identified by means of Monte Carlo (MC) simulations in the Grand Canonical (GC) ensemble. Observables of a GCMC simulation are the system energy *E*, varying as a consequence of the dynamics, and the particle number *N*, varying as particles can be inserted or removed from the simulation box. Simulations were characterized by a maximum number of particles *N*_*max*_ allowed in the simulation box. An MC step was defined as *N*_*max*_ MC moves, where the moves used were the insertion/deletion of a particle, attempted with probability 0.01, and a single particle rototranslational (RT) move, i.e., the contemporary translation and rotation of a single particle^68^, attempted with probability 0.99^69^. The maximum translation length (0.05) and maximum rotation angle (0.1) were set to have an average acceptance rate of the RT move ~0.3 when the model was near the critical point. The average acceptance rate was higher in the dilute phase and lower in the dense phase. The simulation box was a cube with linear size *L* = 8. More details are given in the SN 2A.

### Identification of the critical point

To identify the critical point for a given set of *u*_EE_, *u*_PP_, and *γ*, numerous short simulations for different values of the temperature *T* and of the chemical potential *μ* were performed, in order to approximately locate the coexistence region. Once the coexistence region was identified, few different values of *T* and *μ* were simulated, using 12 independent GCMC simulations per state point. Each simulation started with *N*_0_ = 180 particles and equilibrated for 2.5 × 10^6^ MC steps, which was verified a posterior to be a sufficiently large equilibration time for all simulations. The total run time was set to 5 × 10^7^ MC steps per simulation. One value of the observables was collected every 10^3^ MC steps, and one configuration was saved every 5 × 10^4^ MC steps, resulting in 47,500 datapoints and 950 configurations per simulation. This procedure resulted in a total of 57 × 10^4^ values of *E* and *N* per state point and 11400 configurations.

At the critical point the probability distribution of the scaling variable ${{{{\mathcal{M}}}}}=N+sE$ is the same (up to second-order corrections that vanish in the thermodynamic limit) as the distribution of the magnetization of the Ising model^70^, where *s* is a fitting parameter with non-universal values. Using data generated simulating the state point (*T*, *μ*) and exploiting the histogram reweighting method^71^, it is possible to identify new values $({T}^{{\prime} },{\mu }^{{\prime} })$, as well as to fit an optimal value of *s*, such that the distribution of ${{{{\mathcal{M}}}}}$, rescaled to have unit variance, matches the Ising magnetization distribution, computed as in ref. ^72^. Numerical simulations have been performed for each (*u*_EE_, *u*_PP_, *γ*) set until the fit of the reweighted distribution of ${{{{\mathcal{M}}}}}$ to the Ising magnetization distribution produced an error lower than 0.140, the error being simply the norm of the difference between the two functions. The resulting values of ${T}^{{\prime} }$ and ${\mu }^{{\prime} }$ have been ascribed to be the critical ones. More details are given in the SN 2B.

## Supplementary information

Supplementary Material

## Data Availability

Data can be made available upon request by simply writing to one the authors.

## Code availability

Data for this study have been produced using the code made available at https://github.com/DaniMuzi/IPPs-critical-point. Together with the C code to produce the data, data analytics tools to reproduce the results presented in this work are available at the same link.
