# Supplementary material for: Liquid-liquid phase separation driven by charge heterogeneity
Source: Commun Phys. 2024 Dec 19;7(1):412. doi: 10.1038/s42005-024-01875-4 (PMC11721519; doi:10.1038/s42005-024-01875-4)
Supplement: Supplementary file 1 — Supplementary Material [file 42005_2024_1875_MOESM1_ESM.pdf]

LIQUID-LIQUID PHASE SEPARATION DRIVEN BY CHARGE HETEROGENEITY
SUPPLEMENTAL MATERIAL

Daniele Notarmuzi[*⋆], Emanuela Bianchi[*†]
[*]*Institut für Theoretische Physik, TU Wien, Wiedner Hauptstraße 8-10, A-1040 Wien, Austria*
†*CNR-ISC, Uos Sapienza, Piazzale A. Moro 2, 00185 Roma, Italy*
[⋆]daniele.notarmuzi@tuwien.ac.at

Numerical simulations of the Inverse Patchy Particle (IPP) model have been performed by adapting the publicly available code published with Ref. [1]. The resulting code, together with data analytics tools to reproduce the results presented in this paper, is available at [2].

Supplementary Note 1. MODEL

As stated in the main text, the interaction energy between a pair of IPPs is

$$U(r,\Omega) = \sum_{\alpha\beta} \epsilon_{\alpha\beta}\omega_{\alpha\beta}(r,\Omega)\ . \tag{S1}$$

We explain the above equation by taking as example the EP configuration shown in Fig. S1, where the two particles are labeled as 1 and 2 and the two patches of particle 2 are labeled as ”Western” (W) and ”Eastern” (E). Note, however, that the distinction is entirely based on the visual representation as the two patches, as well as the two particles, are identical in the model. In Eq. (S1) the symbols α and β specify the interaction sites, i.e., α and β can be either the central (c) site or one of the off-center (oc) sites. For example, α could represent the central site of particle 1, $\alpha = c$, and β could represent one of the two off-center sites of particle 2, say the Western site, $\beta = W$, where W stands for ”Western”. $\epsilon_{\alpha\beta}$ characterizes the energy strength of the $\alpha\beta$ interaction type. Hence, in the example, $\epsilon_{\alpha\beta} = \epsilon_{cW}$ represents the interaction strength between the center site of particle 1 and the Western site of particle 2. Note, however, that all particles are identical so (i) the indices 1 and 2 of the particle can be exchanged and (ii) it is not relevant which patch one is considering: $\epsilon_{\alpha\beta}$ are constants that uniquely define a model and they do not depend on the orientation Ω nor on the distance r between particles. Finally, $\omega_{\alpha\beta}$ takes into account the weight of the $\alpha\beta$ contribution to the total pair energy and thus depends on r and Ω. Specifically, the $\omega_{\alpha\beta}$s are chosen to be proportional to the relative overlap volume between pairs of interaction spheres pertaining the selected $\alpha\beta$ interaction type for the given particle configuration [3].

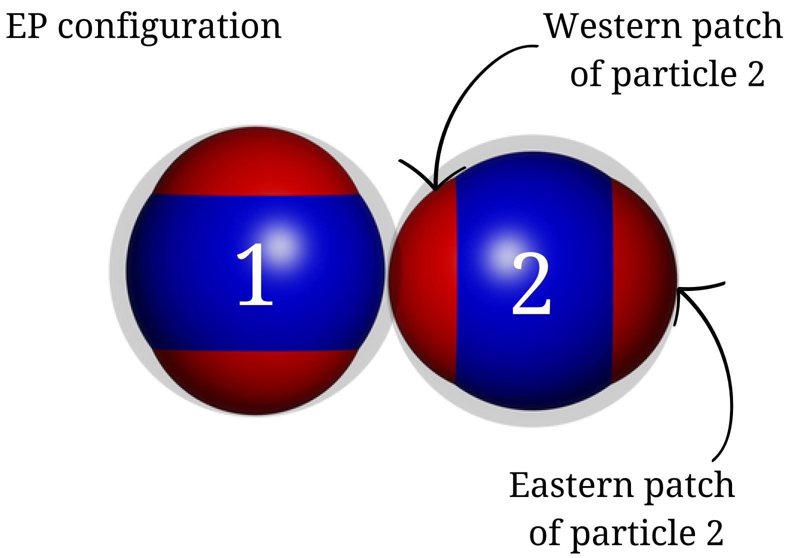

Figure S1. The EP reference configuration. White numbers on each particle label the particles. To facilitate the discussion, the patches associated to the off-center sites of particle 2 are named “Western” (W) and “Eastern” (E).

Hence, in the usual example, ω_{cW} is the relative overlap volume between the interaction sphere associated to the center site of particle 1, whose radius is $\sigma_c + \delta/2$, and the interaction sphere associated the Western site of particle 2, whose radius is

$\sigma_p = \sigma_c + \delta/2 - a$. By relative overlap volume we mean that the overlap volume between the two spheres is normalized by its maximum possible value, so that $0 \leq \omega_{\alpha\beta} \leq 1$. The maximum possible value is the total volume of the smallest of the two spheres, which in the example is the one with radius σ_p. Note that in the calculation of $\omega_{\alpha\beta}$ it does matter which particle is labeled as 1 and which is labeled as 2, as well as it does matter which off-center site of particle 2 is considered. In the example considered in Fig. S1 the overlap volume $\omega_{cW} > 0$, while $\omega_{cE} = 0$, where E stands for the Eastern site of particle 2, as the Eastern patch of particle 2 does not overlap with the equatorial region of particle 1.

Overall, in the sum over α and β appearing in Eq. (S1) all possible combinations of sites are considered, so that the calculation of U requires to compute the relative overlap volume between the equatorial regions of both particles (one term of the form (α,β) = (c,c)), between the equatorial region of one particle with the two patches of the other particle (two terms of the form (α,β) = (c, oc) and two of the form (α,β) = (oc, c)) – and between the patches of one particle with the patches of the other particle (four terms of the form (α,β) = (oc,oc)).

The $\epsilon_{\alpha\beta}$-values can be fixed by mapping the coarse-grained model to an orientation-dependent DLVO-like description [3, 4]. The DLVO-like potential, in turn, explicitly depends on the amount of charge located at the interaction sites. It follows that the values of $\epsilon_{\alpha\beta}$ are directly related to the amount of charge of the sites. Specifically, one could assign the values of $\epsilon_{\alpha\beta}$ by explicitly considering a given physical system, assign the screening conditions, calculate the DLVO-like potential and then opportunely tune the values of $\epsilon_{\alpha\beta}$ so to map the potential of Eq. (S1) onto the DLVO-like potential provided by the mean-field theory. Once the array $\boldsymbol{\epsilon} = (\epsilon_{c,c}, \epsilon_{c,oc}, \epsilon_{oc,oc})$ is known, the energy of a pair configuration AB is calculated as

$$u^{AB} = w^{AB}_{c,c}\epsilon_{c,c} + w^{AB}_{c,oc}\epsilon_{c,oc} + w^{AB}_{oc,oc}\epsilon_{oc,oc} \tag{S2}$$

where the $w^{AB}_{\alpha\beta}$ are the *total* overlap volumes between all the $\alpha\beta$ sites given the AB configuration. These AB configurations are the reference configurations EE, EP, PP used in the main text and are chosen so that the interaction energy U is dominated by the center/center (c,c), center/off-center (c,oc) and off-center/off-center (oc,oc) interaction respectively. Taking again the example of the EP configuration in Fig. S1, the matrix element $w^{EP}_{c,oc}$ is the sum of the overlap volumes between the interaction sphere associated to the center of particle 1 and the interaction spheres associated to both the off-center sites of particle 2, plus the vice-versa (the same overlap volumes but with particle labels exchanged), under the condition that, the mutual orientation between the two particles is specified by the EP configuration. Once the array $\boldsymbol{\epsilon}$ is assigned, the array $\mathbf{u}$ is hence calculated as

$$\begin{aligned} u^{EE} &= w^{EE}_{c,c}\epsilon_{c,c} + w^{EE}_{c,oc}\epsilon_{c,oc} + w^{EE}_{oc,oc}\epsilon_{oc,oc} \\ u^{EP} &= w^{EP}_{c,c}\epsilon_{c,c} + w^{EP}_{c,oc}\epsilon_{c,oc} + w^{EP}_{oc,oc}\epsilon_{oc,oc} \\ u^{PP} &= w^{PP}_{c,c}\epsilon_{c,c} + w^{PP}_{c,oc}\epsilon_{c,oc} + w^{PP}_{oc,oc}\epsilon_{oc,oc}. \end{aligned} \tag{S3}$$

As in the present work we want to systematically address the role played by electrostatic interactions, we rather assign the array $\mathbf{u}$ and compute the array $\boldsymbol{\epsilon}$ by solving the following system of equations

$$w^{-1}\mathbf{u} = \boldsymbol{\epsilon}, \tag{S4}$$

where w^{-1} is the inverse of the matrix w defined as

$$\begin{vmatrix} w^{EE}_{c,c} & w^{EE}_{c,oc} & w^{EE}_{oc,oc} \\ w^{EP}_{c,c} & w^{EP}_{c,oc} & w^{EP}_{oc,oc} \\ w^{PP}_{c,c} & w^{PP}_{c,oc} & w^{PP}_{oc,oc} \end{vmatrix} . \tag{S5}$$

Once $\boldsymbol{\epsilon}$ is determined via Eq. S4, the pair energy U can be computed at any inter-particle distance and mutual orientation.

We note that the choice of the functions $\omega_{\alpha\beta}$ is completely arbitrary. In principle, any function that depends on r and Ω can be used to weight the energetic contributions $\epsilon_{\alpha\beta}$. This arbitrary choice, however, guarantees a good match between the potential U and the DLVO-like potential derived analytically [3].

Supplementary Note 2. METHODS

A. Grand Canonical Monte Carlo simulations

The critical points displayed in the main text have been identified by means of Monte Carlo (MC) simulations in the Grand Canonical (GC) ensemble. Observables of a GCMC simulation are the system energy E, varying as a consequence of the dynamics, and the particle number N, varying as particles can be inserted or removed from the simulation box. Simulations were characterized by a maximum number of particles N_{max} allowed in the simulation box. A MC step was defined as N_{max} MC moves, where the moves used were the insertion/deletion of a particle, attempted with probability 0.01, and a single particle rototranslational (RT) move, i.e., the contemporary translation and rotation of a single particle [1]. The latter move was attempted with probability 0.99. The maximum translation length (0.05) and maximum rotation angle (0.1) have been set to have an average acceptance rate of the RT move around 0.3 when the model was near the critical point. The average acceptance rate was higher in the diluted phase and lower in the dense phase. The simulation box was a cube with linear size $L = 8$.

B. Identification of the critical point

To identify the critical point for a given set of u_{EE}, u_{PP} and γ, numerous short simulations for different values of the temperature T and of the chemical potential μ have been performed, in order to approximately locate the coexistence region. Once the coexistence region was identified, few different values of T and μ have been simulated, using 12 independent GCMC simulations per state point. Each simulation started with $N_0 = 180$ particles and equilibrated for $2.5 \cdot 10^6$ MC steps, which was verified a posterior to be a sufficiently large equilibration time for all simulations. The total run time was set to $5 \cdot 10^7$ MC steps per simulation. One value of the observables was collected every 10^3 MC steps and one configuration was saved every $5 \cdot 10^4$ MC steps, resulting in 47500 datapoints and 950 configurations per simulation. This procedure resulted in a total of $57 \cdot 10^4$ values of E and N per state point and 11400 configurations.

At the critical point the the probability distribution of the scaling variable $\mathcal{M} = N + sE$ is the same (up to second order corrections that vanish in the thermodynamic limit) of the distribution of the magnetization of the Ising model [5], where s is a fitting parameter with non universal values. Using data generated simulating the state point (T, μ) and exploiting the histogram reweighting method [6], it is possible to identify new values of (T', μ'), as well as to fit an optimal value of s, such that the distribution of $\mathcal{M}$, rescaled to have unit variance, matches the Ising magnetization distribution, computed as in Ref. [7]. Numerical simulations have been performed for each $(u_{\mathrm{EE}}, u_{\mathrm{PP}}, \gamma)$ set until the fit of the reweighted distribution of $\mathcal{M}$ to the Ising magnetization distribution produced an error lower than 0.140, where the error is simply the norm of the difference between the two functions. The resulting values of T' and μ' have been ascribed to be the critical ones.

Fig. S2 exemplifies the result of the procedure described in the materials and methods section of the main text for the identification of the critical point. The system considered in the figure is $u_{\mathrm{EE}} = u_{\mathrm{PP}} = 0.0$ and $\gamma = 50°$. Simulations have been performed at the state point $(T, \mu) = (0.1286, -0.427)$. The figure shows the original distribution of N and E as obtained from the simulations and the reweighted distributions associated to the critical parameters $(T_c, \mu_c) = (0.1285, -0.428)$. The values of T_c and μ_c have been obtained by matching the distribution of $\mathcal{M}$ to the Ising distribution, as shown in the figure. The fit further provided the optimal value $s = 0.25$.

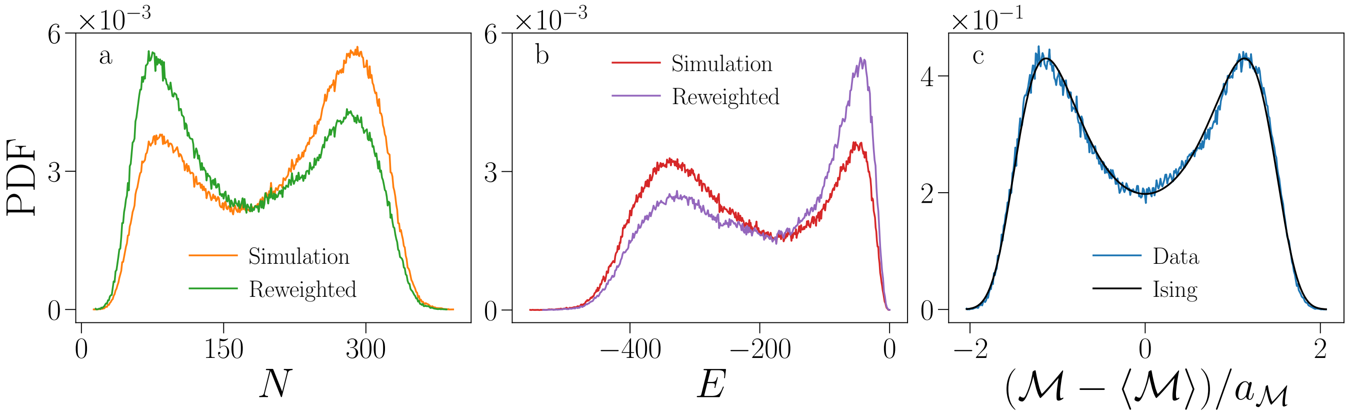

Figure S2. Critical distributions before and after reweighting for $u_{\mathrm{EE}} = u_{\mathrm{PP}} = 0.0$ and $\gamma = 50°$. (a) The distribution of the particle number N as obtained from the simulation and after reweighting. (b) Same as in (a), but for the energy distribution. (c) The distribution of the scaling variable $\mathcal{M}$, compared to the Ising magnetization distribution. The variable $a_{\mathcal{M}}$ has been chosen for the distribution to have unit variance.

C. Simulations at the critical point

The procedure explained above allows to infer the critical parameter T_c and μ_c without performing a numerical simulation exactly at $T = T_c$ and $\mu = \mu_c$. The histogram reweighting technique further allows to compute the critical density ρ_c and the critical energy density u_c: inferring T_c and μ_c automatically provides with the joint particle number - energy distribution $P(N, E)$. The latter can be then marginalized to finally obtain $\rho_c = \int_N P(N')dN'/V$ and the critical energy density $u_c = \int_E P(E')dE'/V$. However, the analysis of the structural properties of the critical phases requires further simulations to be performed, which must be sufficiently fine tuned to effectively allow for the samples to represent critical behaviour.

A simple way to verify whether a simulation is sufficiently fine tuned follows from the scaling properties associated to the critical point. In particular, if a simulation is performed at the critical point, then the distribution of the variable $\mathcal{M}$ must coincide with the Ising magnetization distribution without the need of using histogram reweighting. The simulations performed to infer the critical point have all been made using four digits precision for the temperature and three digits precision for the chemical potential. Hence, it is natural to study the critical configurations by using the same number of digits. As stated, the critical point

inferred for $u_{\rm EE} = u_{\rm PP} = 0.0$ and $\gamma = 50°$ is $(T_c, \mu_c) = (0.1285, -0.428)$. Performing numerical simulations with these settings provide with the results shown in Fig. S3, where the distribution of N and E were used to compute the distribution of $\mathcal{M}$ without reweighting, only allowing for the parameter s to be optimized again. The match between the distribution of $\mathcal{M}$ and the Ising magnetization distribution is clearly not good. It follows that the simulation can not be considered at the critical point and the configurations obtained can not be used to compute the average functionality.

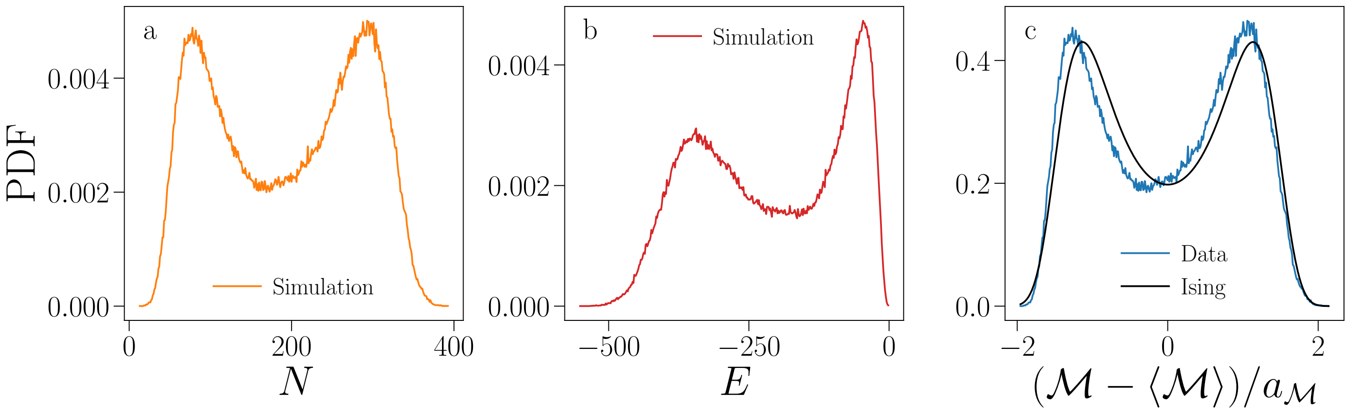

Figure S3. Tentatively critical distributions for $u_{\rm EE} = u_{\rm PP} = 0.0$ and $\gamma = 50°$. (a) The distribution of the particle number N as obtained from the simulation at $(T_c, \mu_c) = (0.1285, -0.428)$. (b) Same as in (a), but for the energy distribution. (c) The distribution of the scaling variable $\mathcal{M}$, compared to the Ising magnetization distribution. The variable $a_{\mathcal{M}}$ has been chosen for the distribution to have unit variance.

The reason why the simulations do not reproduce the correct scaling properties of the critical point is that the thermodynamic parameters were not fine tuned enough. The fact that a good fine tuning is required for a system to truly display critical behavior is a natural consequence of the divergence of the susceptibility at the critical point: as the system is extremely susceptible, a small change in the thermodynamic parameters can lead to large changes in the model's behaviour. Hence, the same simulations were performed again, but using more digits, so to better tune the system at criticality. Results for $(T, \mu) = (0.128519479875, -0.428066275416)$ are shown in Fig. S4. As in the case of Fig. S3, the variable $\mathcal{M}$ has been computed without reweighting and only allowing the parameter s to vary, obtaining again $s = 0.25$. The configurations sampled in these simulations can be used to compute the average functionality. As for the determination of critical points, 12 parallel simulations were run, each with run time set to $5 \cdot 10^7$ MC steps per simulation. One value of the observables N and E was collected every 10^3 MC steps and a configuration was saved every $5 \cdot 10^4$ MC steps.

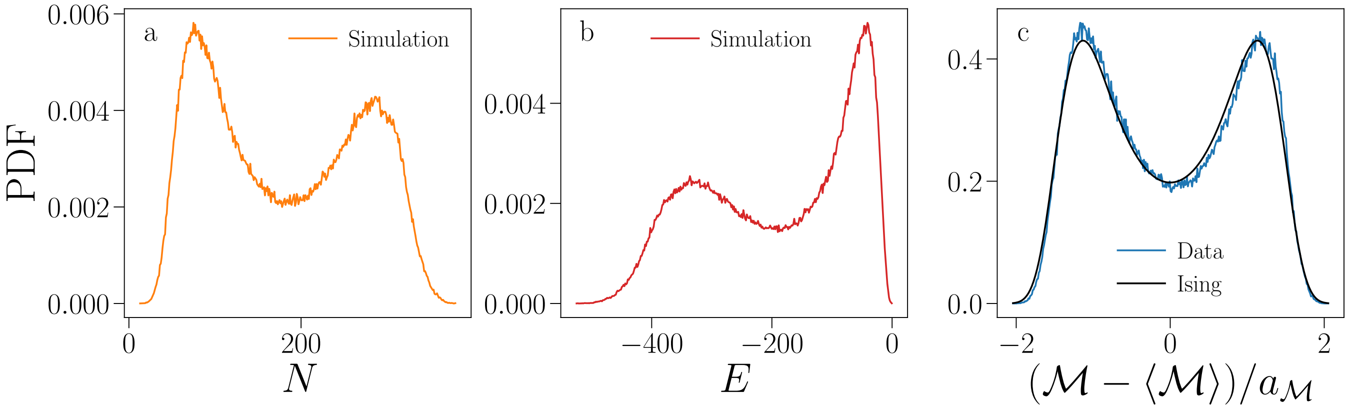

Figure S4. Critical distributions for $u_{EE} = u_{PP} = 0.0$ and $\gamma = 50°$. (a) The distribution of the particle number N as obtained from the simulation at $(T, \mu) = (0.128519479875, -0.428066275416)$. (b) Same as in (a), but for the energy distribution. (c) The distribution of the scaling variable $\mathcal{M}$, compared to the Ising magnetization distribution. The variable $a_{\mathcal{M}}$ has been chosen for the distribution to have unit variance.

Supplementary Note 3. ANALYSIS

A. Compact vs branched structures

Compact clusters have smaller radius of gyration if compared to branched clusters of the same size. The radius of gyration of a given cluster of size s can be computed as

$$R_g = \frac{1}{s}\sum_i |\mathbf{r_i} - \mathbf{r_{cdm}}|^2 \tag{S6}$$

where $\mathbf{r_{cmd}}$ is the center of mass of the cluster. Stauffer and Aharony [8] define three different ways to average R_g over the whole system, i.e.,

$$\xi_k = \frac{\sum_s \langle R_g^2 \rangle_s s^k n_s}{\sum_s s^k n_s} \quad \text{with} \quad k = 0, 1, 2 \tag{S7}$$

where n_s is the number of clusters of size s and the symbol $\langle \cdot \rangle_s$ implies that the average is done over clusters of size s.

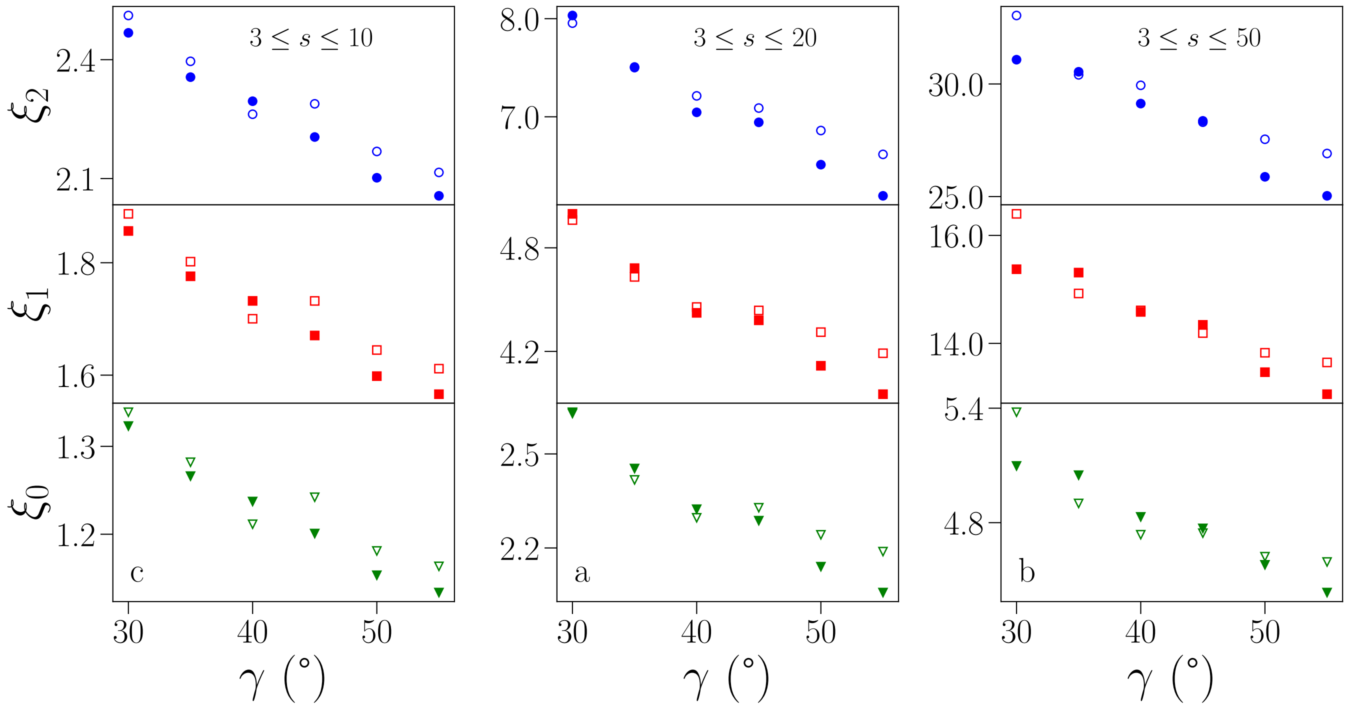

Figure S5. System averaged radius of gyration as defined in Eq. (S7). Blue circles in the top panels show ξ_2, red squares in the middle panels show ξ_1 and green triangles in the lower panels show ξ_0. Filled markers represent data for $u_{EE} = u_{PP} = 0.0$ (IPP$_{ro}$), empty markers are for $u_{EE} = 0.5$, $u_{PP} = 2.0$ (IPP$_{ref}$). (a) The summation in Eq. (S7) ranges from $s = 3$ to $s = 10$. (b) Same as in (a), but the summation in Eq. (S7) is from $s = 3$ to $s = 20$. (c) Same as in (a), but the summation in Eq. (S7) is from $s = 3$ to $s = 50$.

Fig. S5 displays ξ_0, ξ_1 and ξ_2 for $u_{EE} = u_{PP} = 0.0$ and $u_{EE} = 0.5$, $u_{PP} = 2.0$. The summation in Eq. (S7) has been bounded to a maximal value of the cluster size as the number of extremely large clusters is not statistically significant, but these clusters would nonetheless dominate the summation, making the measure of ξ too noisy. For both the systems considered ξ_k is a monotonically decreasing function of γ, regardless of k. Furthermore, data for the repulsive system $u_{EE} = 0.5$, $u_{PP} = 2.0$ (IPP$_{ref}$) tend to display larger values of ξ_k if compared with the data for the non-repulsive system $u_{EE} = u_{PP} = 0.0$ (IPP$_{ro}$) at the same γ. These results are perfectly consistent with the behaviour observed for the critical density ρ_c: models characterized by large ρ_c tend to have large ξ_k and vice versa, confirming the interpretation given in the main text, i.e., that the behaviour of the density can be explained in terms of the compactness of the clusters. Compact cluster have indeed a smaller radius of gyration and the measure of ξ confirms that ρ_c is large for systems where compact structures are common. Fig. S6 shows examples of branched trimers for $\gamma = 30°$ and compact trimers for $\gamma = 55°$, clarifying why the gyration radius of clusters with large γ tend to be smaller.

In addition to the measure of ξ_k, we calculate $\langle R_g^2 \rangle_s$ and show it as a function of the cluster size s in Fig. (S7): for both the IPP$_{ro}$ and the IPP$_{ref}$ systems, larger values of $\langle R_g^2 \rangle_s$ are systematically sampled in systems of IPPs with small patches (which

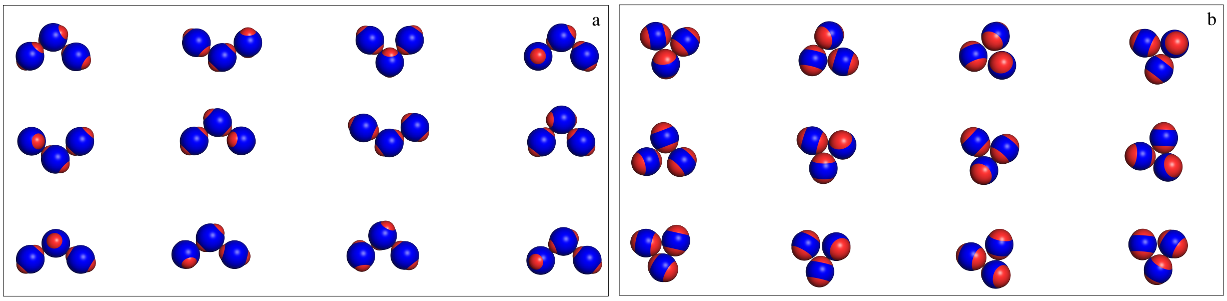

Figure S6. Compact vs branched trimers at criticality for $u_{\mathrm{EE}} = u_{\mathrm{PP}} = 0.0$ ($\mathrm{IPP_{ro}}$). (a) Typical configurations of branched trimers for $\gamma = 30°$. Samples from simulations at the inferred critical point. (b) Typical configurations of compact trimers for $\gamma = 55°$. Samples from simulations at the inferred critical point.

is especially true at large values of s), confirming that on increasing γ clusters become more compact. Moreover, $\langle R_g^2 \rangle_s$ of the $\mathrm{IPP_{ref}}$ system at a given γ assumes slightly larger values with respect to the $\langle R_g^2 \rangle_s$ of the respective $\mathrm{IPP_{ro}}$ system at the same γ, confirming that also the electrostatic repulsion plays a role in the compactness of the clusters: on increasing the directional repulsion, critical configurations are characterized by the formation of increasingly less compact clusters.

Together with the measure of $\langle R_g^2 \rangle$, Fig. (S7) shows four different critical configurations of $\mathrm{IPP_{ro}}$ systems, two for $\gamma = 30°$ and two for $\gamma = 55°$. For both systems, we show a configuration of the dilute phase and one of the dense phase to visually convey how the two phases strongly differ at the microscopic level: the dense fluid is characterized by a unique, large cluster, while the dilute fluid is made of small, non interacting clusters.

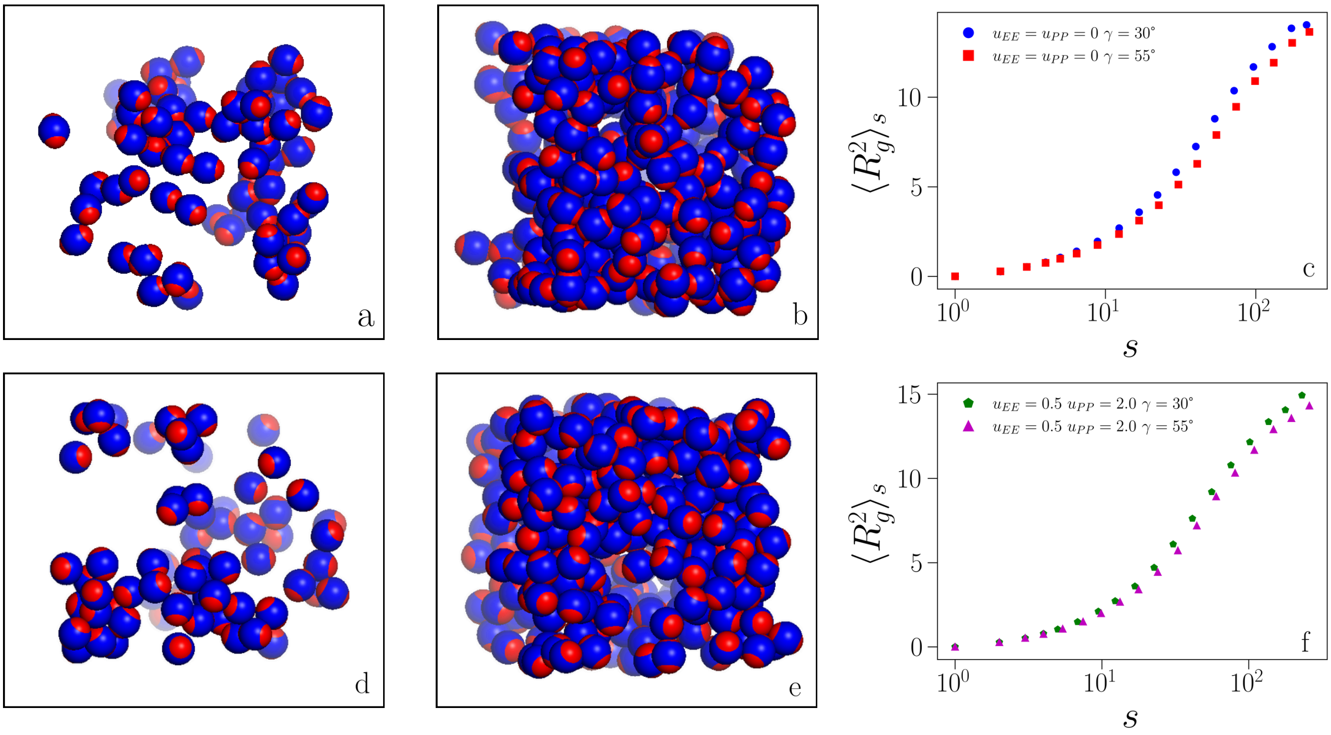

Figure S7. Configurations at the critical point: snapshot of the $\mathrm{IPP_{ro}}$ system with $\gamma = 30°$ (a) in the dilute phase ($N = 70$ particles) and (b) in the dense phase ($N = 280$ particles); snapshot of the $\mathrm{IPP_{ro}}$ system with $\gamma = 55°$ (d) in the dilute phase ($N = 70$ particles) and (e) in the dense phase ($N = 300$ particles). Average radius of gyration as a function of the cluster size for (c) two $\mathrm{IPP_{ro}}$ and (f) two $\mathrm{IPP_{ref}}$ systems with different γ values as labeled.

B. Bonding volume

The bonding volume is defined as

$$V_b = 4\pi \int_{2\sigma_c}^{2\sigma_c+\delta} S(r) r^2 dr \tag{S8}$$

where $S(r)$ is the average fraction of solid angle available to bonding [9]. To compute $S(r)$, a pair of particles was simulated, one being fixed in position and orientation, while the other being assigned a random orientation and a random position in the shell of radii $2\sigma_c$ and $2\sigma_c + \delta$. This range of distances ensures the existence of a random geometric bond. $S(r)$ is then computed as the fraction of configurations in which a geometric bond is also an energetic bond, averaged at different values of r.

In Fig. S8 the bonding volume is shown, together with its second derivative w.r.t. γ.

Consistently with the behaviour of the critical temperature reported in the main text, V_b is found to monotonically increase with γ. Furthermore, V_b decreases as electrostatic repulsion grows, with u_{EE} having more effect than u_{PP}. It is worth noting that when parameters gradually change, the curvature of T_c as a function of γ changes continuously from positive to negative as u_{EE} grows, while it diminishes as u_{PP} grows but does not change sign, see Fig.2d of the main text. The same happens to the curvature of V_b, expressed by its second derivative, which decreases with γ – quickly with u_{PP} changes, slowly u_{EE} changes – and it can change sign with γ. For $\gamma = 30$ the dependence of V_b on u_{PP} is rather weak, as it is for T_c but, curiously, V_b remains a (slightly) decreasing function of u_{PP}, while T_c slowly increases with u_{PP}.

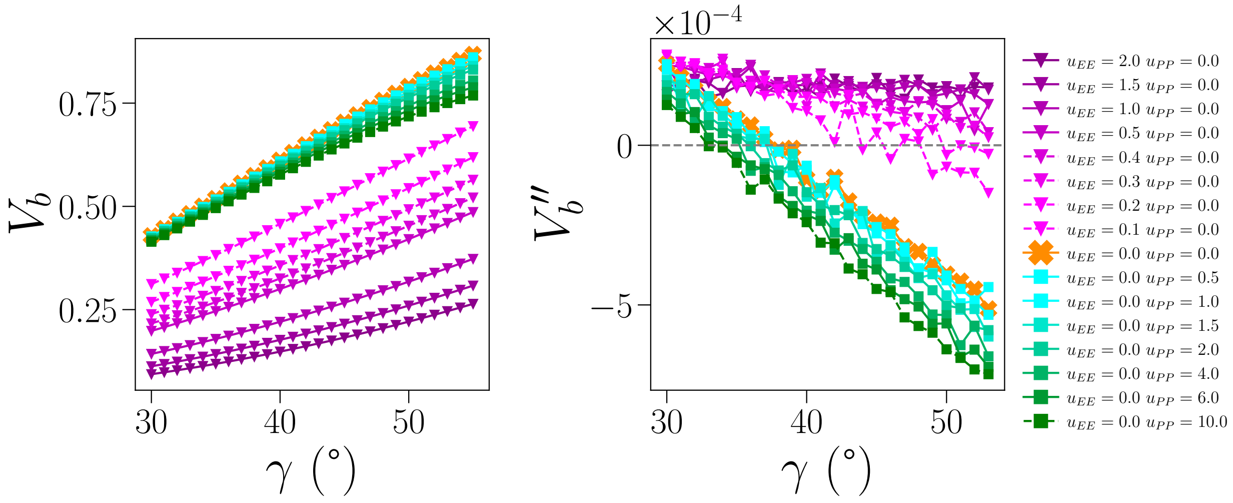

Figure S8. Bonding volume V_b and its second derivative w.r.t. γ for different interaction strengths. Orange crosses are the data corresponding to $u_{\mathrm{EE}} = u_{\mathrm{PP}} = 0$ (IPP$_{\mathrm{ro}}$). The color goes from magenta to purple as u_{EE} grows and from cyan to green as u_{PP} grows. Curves referring to values of u_{EE} and u_{PP} for which we computed the critical point are shown as solid lines, otherwise the line is dashed.

Supplementary References

[1] L. Rovigatti, J. Russo, and F. Romano, The European Physical Journal E **41** (2018).
[2] "Code to reproduce the results," `https://github.com/DaniMuzi/IPPs-critical-point`.
[3] E. Bianchi, G. Kahl, and C. N. Likos, Soft Matter **7**, 8313 (2011).
[4] M. Stipsitz, E. Bianchi, and G. Kahl, J. Chem. Phys. **142**, 114905 (2015).
[5] A. D. Bruce and N. B. Wilding, Phys. Rev. Lett. **68**, 193 (1992).
[6] A. M. Ferrenberg and R. H. Swendsen, Phys. Rev. Lett. **61**, 2635 (1988).
[7] M. M. Tsypin and H. W. J. Blöte, Phys. Rev. E **62**, 73 (2000).
[8] D. Stauffer and A. Aharony, *Introduction To Percolation Theory: Second Edition (2nd ed.)* (Taylor & Francis, London, 1992).
[9] F. Sciortino, E. Bianchi, J. F. Douglas, and P. Tartaglia, The Journal of Chemical Physics **126**, 194903 (2007).
